# Cefotaxime-Resistant *Salmonella enterica* in Travelers Returning from Thailand to Finland

**DOI:** 10.3201/eid2007.131744

**Published:** 2014-07

**Authors:** Marianne Gunell, Laura Aulu, Jari Jalava, Susanna Lukinmaa-Åberg, Monica Österblad, Jukka Ollgren, Pentti Huovinen, Anja Siitonen, Antti J. Hakanen

**Affiliations:** National Institute for Health and Welfare, Turku, Finland (M. Gunell, J. Jalava, M. Österblad, P. Huovinen, A.J. Hakanen);; National Institute for Health and Welfare, Helsinki, Finland (L. Aulu, S. Lukinmaa-Åberg, J. Ollgren, A. Siitonen);; University of Turku, Turku, (M. Gunell, P. Huovinen, A.J. Hakanen)

**Keywords:** *Salmonella enterica*, 4,[5],12:i:-, cefotaxime, resistant, *qnr*, Thailand, travelers, Finland, enteric infections

## Abstract

During 1993–2011, cefotaxime resistance among *Salmonella enterica* isolates from patients in Finland increased substantially. Most of these infections originated in Thailand; many were *qnr* positive and belonged to *S*. *enterica* serovar Typhimurium and *S. enterica* monophasic serovar 4,[5],12:i:-. Although cefotaxime-resistant salmonellae mainly originate in discrete geographic areas, they represent a global threat.

*Salmonella* spp. are a common cause of foodborne illnesses globally, but illnesses caused by *Salmonella* infections vary from mild diarrhea (travelers’ diarrhea) to severe generalized infections (*1*). Certain *Salmonella* serotypes are more commonly linked to human infections and for example, the monophasic 4,[5],12:i:- variant of *S. enterica* serovar Typhimurium has caused an increasing number of *Salmonella* infections in humans during the last decade (*2*). Antimicrobial agents, usually fluoroquinolones and extended-spectrum cephalosporins, are needed for the treatment of patients with invasive *Salmonella* infections (*3*).

The abundant use of antibiotics in human and veterinary medicine and in food production has led to antimicrobial drug resistance (*4*), and the numbers and proportions of extended-spectrum β-lactamase (ESBL)– and AmpC β-lactamase–producing strains of *Enterobacteriaceae* have increased worldwide (*3*,*5*–*7*). Although reduced fluoroquinolone susceptibility among *S. enterica* isolates has increased since the late 1990s (*8*,*9*), *Salmonella* spp. have remained cephalosporin-susceptible. Coexistence of ESBL and plasmid-mediated quinolone resistance genes in *Salmonella* and in other *Enterobacteriaceae* genera have been reported and there are existing reports on extended-spectrum cephalosporin-resistant and ESBL-producing *Salmonella* isolates (*3*,*4*,*10*).

To date, *Salmonella* isolates that have acquired resistance determinants against fluoroquinolones and extended-spectrum cephalosporins have been reported only anecdotally in Finland. This study describes a systematic analysis of extended-spectrum cephalosporin–resistant *Salmonella* isolates in Finland during a 19-year period.

## The Study

During 1993–2011, 43,171 *S. enterica* isolates were sent to the National Salmonella Reference Centre of the National Institute for Health and Welfare (THL) in Finland. This *Salmonella* collection contains ≈85% (range 75.9%–91.1%) of all *Salmonella* isolates collected annually in Finland during the study period. All of these isolates were screened for cefotaxime susceptibility (*11*). A total of 225 cefotaxime-nonsusceptible *S. enterica* isolates were identified; 183 of these, collected during 2000–2011, were genotyped. The isolates were screened and serotyped in the Bacteriology Unit at THL.

We confirmed phenotypic ESBL using disk diffusion tests (*11*). Cefotaxime-nonsusceptible isolates were screened for the ESBL genes TEM, SHV, and CTX-M by PCR (*7*). CTX-M–positive *Escherichia coli*, SHV-positive *Klebsiella pneumoniae*, and TEM-positive *E. coli* were used as positive ESBL controls. Isolates having only a TEM determinant were further classified by pyrosequencing (*12*).

We also screened the cefotaxime-nonsusceptible isolates for AmpC production. PCR was used to amplify the AmpC β-lactamase genes CMY, FOX, DHA, ACC, MOX, and EBC by using previously described primers (*13*). The AmpC multiplex-PCR reaction (50 µL) consisted of 0.2 pmol/µL of each primer, 0.06 U/µL AmpliTaq Gold DNA polymerase, 5 µL AmpliTaq Gold buffer, 2 mM MgCl_2_, and 0.2 mM dNTP mix (Life Technologies Europe, Espoo, Finland). The PCR program consisted of an initial denaturation at 94°C for 10 minutes, then 38 cycles of DNA denaturation at 94°C for 30 seconds, primer annealing at 64°C for 30 seconds, and extension at 72°C for 1 minute.

We determined susceptibility to the antimicrobial drugs ciprofloxacin, nalidixic acid, and meropenem using the standard agar dilution method according to the Clinical Laboratory and Standards Institute guidelines (*11*). We screened isolates showing reduced fluoroquinolone susceptibility; specifically, to ciprofloxacin (MIC ≥0.125 µg/mL), that were susceptible or resistant on a low level to nalidixic acid (MIC ≤32 µg/mL) (*9*) for transferable plasmid-mediated quinolone resistance determinants. We screened the *qnrA*, *qnrB*, *qnrS*, and *aac(6′)-Ib-cr* genes with a previously described method (*14*).

We performed the statistical analysis using a log-binomial model and year as an explanatory variable to assess the log-linear trend in time in the percentage/proportion of cefotaxime-nonsusceptible *S. enterica* isolates. A p value <0.05 was considered significant. Statistical analyses were performed by using IBM SPSS Statistics Version 21 (IBM Corporation, Armonk, NY, USA).

**Figure 1:**
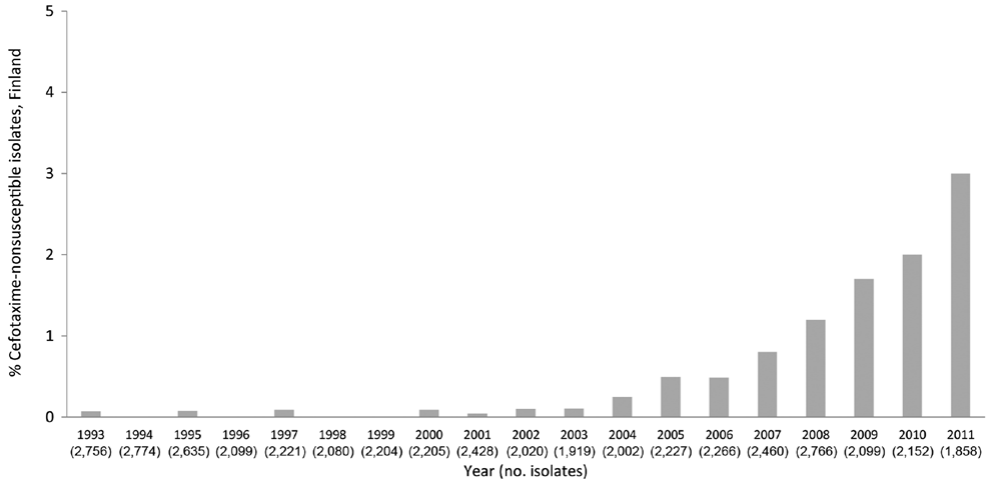
The increasing trend (p<0.001) in the proportion (%) of cefotaxime-nonsusceptible (30-µg disk diameter ≤22 mm) *Salmonella enterica* isolates in Finland during 1993–2011.

During 1993–2011, we found 225 cefotaxime-nonsusceptible *S. enterica* isolates and observed a significantly increasing trend (p<0.001) of cefotaxime-nonsusceptible *S. enterica* isolates (Figure 1). During 1993–1999, 6 *S. enterica* isolates showed nonsusceptibility to cefotaxime. From the year 2000 onwards, cefotaxime-nonsusceptible isolates were detected more frequently, and in the mid-2000s, the absolute number as well as the proportion of cefotaxime-nonsusceptible *Salmonella* isolates started to increase rapidly: 55 (2.96%) of 1,858 isolates were positive for this resistance phenotype in 2011 (Figure 1).

**Figure 2:**
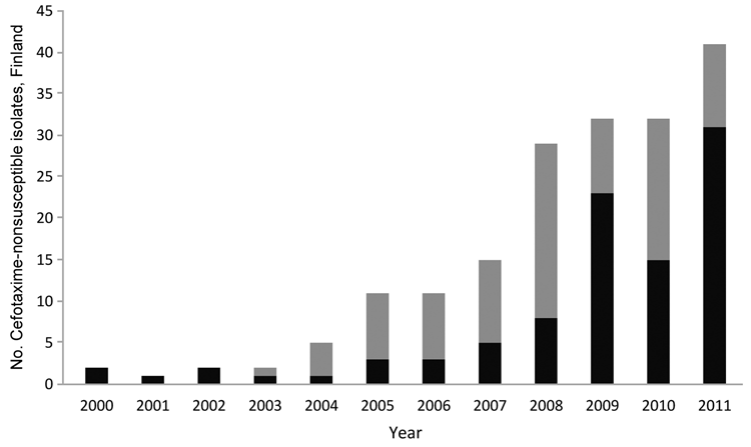
Number of cefotaxime-nonsusceptible *S. enterica* isolates carrying extended-spectrum β-lactamase (black bars) and AmpC genes (gray bars) in Finland, 1993–2011.

During 2000–2011, of the 183 cefotaxime-nonsusceptible isolates, 95 produced ESBL and 88 produced AmpC. The number and proportion of ESBL- and AmpC-positive isolates varied and the number of cefotaxime-nonsusceptible isolates increased (Figure 2). The number of AmpC-positive *S. enterica* isolates was highest in 2008, and the number of ESBL-positive isolates was highest in 2011. During 2000–2005, 10 ESBL-positive isolates were found; these isolates had been identified in samples collected from travelers from Finland returning from the Mediterranean area, Egypt, and European countries. Isolates positive for the SHV gene mainly originated from Egypt. From 2006 onwards, the main geographic origin of ESBL-positive isolates was Southeast Asia; 61% (52/85) of the ESBL isolates originated from Thailand. During the same time, the CTX-M determinant (72/85 isolates) became more common than SHV. Of the ESBL positive isolates, 44 of 95 belonged to *S*. *enterica* ser. Typhimurium or the monophasic 4,[5],12:i:- variant of this serovar; 38 of these originated from Thailand.

AmpC-positive isolates were found from 2003 onwards. During 2003–2004, the AmpC-positive isolates were found in travelers from Finland returning from Spain, India, Mexico, and Africa. From 2005 onwards, the AmpC-positive isolates also commonly originated from Thailand (61/83 isolates). The most common AmpC gene was CMY. Of the AmpC positive isolates, 21 of 88 belonged to *S*. *enterica* ser. Typhimurium or a monophasic 4,[5],12:i:- variant of *S. enterica* ser. Typhimurium serotypes; 8 of these originated from Thailand.

Of the 183 cefotaxime-nonsusceptible *Salmonella* isolates, 47 had the *qnr* phenotype; i.e., they showed reduced susceptibility to ciprofloxacin (MIC ≥0.125 µg/mL) but were susceptible or only resistant on a low level to nalidixic acid (MIC ≤32 µg/mL). These isolates were collected from travelers during 2006–2011. Co-resistance to ESBL determinants were detected in 37 isolates: 35 isolates were CTX-M+*qnr*–positive, including 1 CTX-M+SHV+*qnr*–positive isolate. Two *Salmonella* isolates were SHV+*qnr* positive. Of the 35 CTX-M+*qnr*–positive isolates, 30 isolates originated from Thailand and 23 of them belonged to the serovar *S. enterica ser. Typhimurium* or a monophasic 4,[5],12:i:- variant of *S. enterica* ser. Typhimurium serovars. Ten isolates with an AmpC phenotype were also *qnr*-positive. Nine of these originated from Southeast Asia and 3 of them were *S.* *enterica* ser.Typhimurium or *S. enterica* ser. 4,[5],12:i:- (Table).

**Table:** β-lactamase and plasmid-mediated quinolone resistance genes linked to origin and serovar in cefotaxime-nonsusceptible *Salmonella enterica* isolates, 2006–2011

Gene profile (no. isolates)	Primary origin (no. isolates)	Serovar(s) (no. isolates)
CTX-M + *qnrS* (32)	Thailand (30)	Typhimurium (5); *S. enterica* B 4,[5],12:i:- (18)
CTX-M + *qnrB* (2)	Spain (1)/India (1)	Grumpensis (1)/ Minnesota (1)
CTX-M + *qnrA* (1)	Ethiopia (1)	Concord (1)
SHV + *qnrS* (1)	Egypt (1)	Heidelberg (1)
SHV + *qnrB* (1)	Germany (1)	Senftenberg (1)
CMY + *qnrS* (8)	Thailand (6)	Rissen (4)
CMY/DHA + *qnrS* (1)	Thailand (1)	*S. enterica* B 4,[5],12:i:- (1)
DHA+ *qnrS* (1)	China (1)	Typhimurium (1)

## Conclusions

In this study, we described a significant increase (p<0.001) in cefotaxime nonsusceptibility among *Salmonella* isolates, collected from patients in Finland during 1993–2011. In *Salmonella* spp., cefotaxime nonsusceptibility is thought to be linked to AmpC-type β-lactamases, and production of ESBLs to be more rare (*3*). According to our results, ESBL and AmpC production (51.9% vs. 48.1%) were equally common among the cefotaxime-nonsusceptible *Salmonella* serovars.

During the study period, a change in the geographic origin of cefotaxime-nonsusceptible *Salmonella* isolates was observed: its predominance in Egypt and the Mediterranean area shifted to Thailand and other Southeast Asian countries. We previously reported that *Salmonella* isolates with the *qnr* phenotype are concentrated in Southeast Asia, mainly Thailand (*9*). In this study, 37 ESBL-positive and 10 AmpC-positive *S. enterica* isolates were also *qnr* positive and 40/47 isolates were from Southeast Asia. These results were in concordance with previous reports: ESBL-producing *Enterobacteriaceae* are commonly isolated from patients returning from Southeast Asia (*15*) and ESBL and plasmid-mediated quinolone-resistance mechanisms are commonly found in the same plasmids in *Enterobacteriaceae* and *Salmonella* (*4*,*6*).

We conclude that cefotaxime-nonsusceptible *Salmonella* isolates are already a threat for travelers to Southeast Asia. Because of the mobile nature of the ESBL and AmpC genes, *qnr* resistance determinants, and increased travel, this is a worldwide threat, and makes the treatment for invasive *Salmonella* infections even more challenging.
